# High-Power Fiber Laser Cutting for 50-mm-Thick Cement-Based Materials

**DOI:** 10.3390/ma13051113

**Published:** 2020-03-02

**Authors:** Youngjin Seo, Dongkyoung Lee, Sukhoon Pyo

**Affiliations:** 1 Department of Mechanical and Automotive Engineering, Kongju National University, Cheonan 31080, Korea; syjvlfry1004@gmail.com; 2 School of Urban and Environmental Engineering, Ulsan National Institute of Science and Technology (UNIST), Ulsan 44919, Korea

**Keywords:** laser cutting, multimode fiber laser, cement-based material, elliptical hole

## Abstract

This experimental research highlights the applicability of laser cutting to cement-based materials using multimode fiber lasers. A 9 kW multimode fiber laser is used, and the experimental variables are the water-to-cement ratio, laser speed, and material compositions such as cement paste, cement mortar and ultra high performance concrete (UHPC). The laser cutting performance on the cement-based materials is investigated in the downward laser direction. The kerf width and penetration depth of the cement-based materials are quantitatively evaluated with the parameters in the surface and cross section of the specimens after the laser cutting. Moreover, the material removal zone of each specimen is compared in terms of the penetration shapes in the cross-sectional view. Based on experimental observations, the interaction mechanism between the laser and cement-based materials is proposed.

## 1. Introduction

The demand for concrete fabrication is steadily increasing, especially in the area of concrete cutting employed in architectural renovation projects involving existing concrete structures, as well as in the demolition of nuclear power plants. Conventional material removal methods, such as waterjet cutting and diamond blade cutting, are still mostly used for concrete processing. Moon et al. [1] reported that the performance to confirm whether the computer-aided design (CAD) based path planner could be fulfilled in the remote control of concrete surface grinding. Skarabis and Stöckert [2] used diamond grinding to optimize the noise emission reduction of concrete pavement surfaces. Mostavi et al. [3] investigated the applicability of using drill cuttings in concrete to produce cost-effective material. Furthermore, the effects of fly ash and silica fume on the compressive strength of concrete were evaluated. Foroutan et al. [4] focused on testing the hypothesis that drill cuttings can be integrated into fine aggregates in the production of concrete. In addition, drill cutting is applicable for use in controlled low strength material (CLSM) as well as similar non-structural applications. Sitek et al. [5] utilized the water jet technique in mending works on thermally affected concrete structures. Although the waterjet cutting method can be used to cut most cement-based materials, the method can be implemented only with limited power, leading to long operation times; in addition, the method generates unwanted contaminated wastewater. On the other hands, the diamond blade cutting method can handle only straight or circular geometries. In addition, diamond blade cutting causes micro-fracturing of the concrete because of vibration, thereby resulting in unnecessary removal of concrete.

Laser-aided manufacturing has been used in various fields, such as automobiles, aerospace, electronics, and semiconductors, because of its several advantages, e.g., ease of keeping the workpiece in the right position, low dust and noise generation, fast speed, and noncontact mode of operation. Especially in laser cutting, a small heat-affected zone can prevent the undesirable deformation of materials. Therefore, the laser cutting method has a high level of efficiency. Furthermore, lasers can provide a high energy density and focus on very small spots by optical manipulation. In addition, the laser cutting method has been actively applied to a wide range of materials such as wood, composites, rubber, and metal. Lee et al. [6] proposed the feasibility of laser cutting of compressed cathodes and anodes for lithium-ion batteries. By analyzing the molten pool and temperature distribution after laser cutting on the anode, laser cutting can be used to predict and prevent defects of thermal stresses occurred during the laser cutting. On the other hand, the boundary between active electrode and current collector was clearly shown in the compressed cathode. However, the aluminum contaminated the active electrode when the compressed cathode was used. Wetzig et al. [7] performed a high power fiber laser cutting of aluminum sheets, electrical sheets, and high strength sheets. Several cutting qualities, such as cutting edge, kerf width, and burr formation were analyzed. Pocorni et al. [8] investigated the morphology of a cut front generated by fiber laser. In addition, high-speed imaging (HSI) was used to evaluate the fluid dynamics of the cut front during the laser cutting. Sun et al. [9] proposed a morphology and physical mechanisms of laser-affected damage in laser ablation for thin glass sheets using picosecond lasers. They classified two types of damage morphology observed in a cross-section of a specimen. It was distinguished to be the damaged region caused by high-density free electrons and the heat-affected zone formed by the heat accumulation. Lee et al. [10] fabricated the Spring Contact Probe (SCP) using laser cutting. Interaction relationships between laser and materials, such as a crater size, material removal zone, ablation depth, ablation threshold, and full penetration, were investigated. Haddadi et al. [11] performed laser cutting on an extruded polystyrene sheet using CO_2_ laser. They found that the width of the heat affected zone (HAZ) decreases when the cutting speed is increased using the maximum laser power and compressed air.

Because of these advantages, various studies have been conducted on the use of lasers to process cement-based materials. Muto et al. [12] used a fiber laser system (YLR-5000 MM, IPG) with fiber optic beam delivery to cut concrete. They used a 4 kW ytterbium multimode continuous wave laser and a 1-km-long fiber optic cable, and focused the laser to a 0.75 mm spot through a 120 mm lens. The thickness of the tested concrete slab was 100 mm, and the scanning speed was 5 mm/s. Their research demonstrated the feasibility of remotely using a laser-based concrete cutting technique by specifying the core diameter of the fiber and the fiber length as parameters. Lee et al. [13] used a 1 kW multimode continuous fiber laser (YLS-1000 MM, IPG) emitting at 1070 nm to evaluate the variation of physical and chemical composition of cement-based materials after the laser cutting performance. In particular, they measured compositional variations using energy-dispersive X-ray spectroscopy to study the compositional change before and after the laser interaction with cement-based materials. Furthermore, they determined the laser parameters that were suitable for the complete cutting of cement-based materials. However, the addition of silica sand, silica fume, and silica powder to cement paste resulted in the degradation of the cut quality during laser beam cutting. Crouse et al. [14] used a 1.2 kW CO_2_ laser and a 1.5 kW diode laser to cut a concrete slab. They measured the number of laser cutting passes required to achieve a concrete penetration depth of 120 mm and determined the penetration depth and kerf width according to the number of laser passes. In addition, they performed a numerical simulation to investigate the effect of laser beam characteristics on kerf convergence during multipass processing. The rectangular beam shape of the diode laser yielded higher transmittance and provided a wider kerf during deep-section concrete cutting. In addition, the kerf formed by a CO_2_ laser experienced a higher conductive loss at the edge of the laser spot and a higher temperature in the central region. Because of this influence, the kerf formed by the CO_2_ laser was narrower and more tapered than that formed by the diode laser. Consequently, a high-power diode laser was considered more suitable than a CO_2_ laser for practical concrete cutting applications. Based on the literature review, it can be concluded that most of the previous studies used less than 4 kW of laser power, and a multi-scan technique is applied to cut thick concrete. Furthermore, the interaction mechanism between laser and cement-based materials has not been tested in detail.

Therefore, laser cutting parameters need to be optimized to develop the laser irradiation technique for the efficient removal of cement-based materials. In addition, an understanding of material removal mechanisms is necessary to control and enhance the performance of laser irradiation on cement-based materials. Thus, this study aims to investigate the feasibility of the laser cutting technique to cement-based materials using multimode fiber lasers. Experimental results are presented to show the effect of laser interaction on cement-based materials. Experimental variations are introduced using the laser scanning speed, water-to-cement ratio, and composition of materials such as cement paste, cement mortar, and ultra high performance concrete (UHPC). Cement-based materials without coarse aggregate were chosen in this research to systematically investigate the effects of various forms of silica-based materials on the laser cutting quality. It should be noted that UHPC is one of the most advanced cement-based materials, and active research has been carried out to explore its material characteristics [15,16,17,18]. A multimode fiber laser is used for the experiment. Furthermore, the surface and cross section of the cement-based materials are observed, and the kerf width and penetration depth of the materials are analyzed. Furthermore, the calculated values obtained using elliptic equations are compared with the measured values to explain the elliptical holes observed in the cement paste series. In addition to these observations, a mechanism for the interaction between the laser and cement-based materials is proposed.

## 2. Raw Materials and Mix Design

In this study, various material compositions were designed to investigate the effect of mechanical reactions on cement-based materials according to the laser cutting speed. Table 1 lists the mix proportions of the cement-based materials used in this study. The materials used in the experiment were ordinary Portland cement, silica sand containing approximately 93 wt % of SiO_2_, undensified silica fume (grade 940U, Elkem) containing approximately 95 wt % of SiO_2_, silica powder containing approximately 98% of SiO_2_ with a median diameter of 3.15 μm, and a polycarboxylate-based superplasticizer with 25 wt % solid content by weight. 

Three representative series of cement-based materials were prepared with a thickness of 50 mm and were named as LP, LM, and LU, indicating the use of cement paste, cement mortar, and UHPC for laser cutting, respectively. Furthermore, the LP and LM series names were suffixed with the water-to-cement ratio; for example, LP 0.4 indicates that a water-to-cement ratio of 0.4 is used. Different combinations of materials were set as variables to investigate their effects on laser cutting. The variables of the LU series were named in terms of the weight ratio of silica sand, silica powder, and silica fume. In addition, a superplasticizer was added to LM 0.25 and the LU series to ensure proper mixing by following previous research [19,20].

## 3. Experiment

**Figure 1:**
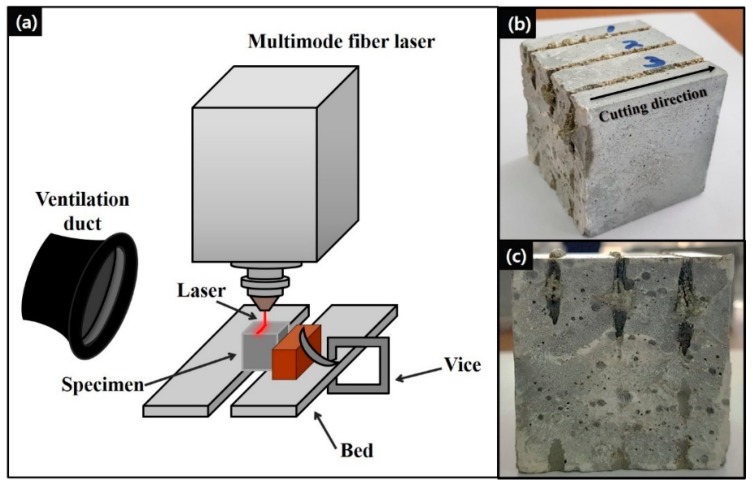
(**a**) Experimental setup, (**b**) bird’s-eye view, and (**c**) front view of concrete specimen after experiment.

In this study, specimens were prepared by a planetary mixer. A fresh mixture was poured into molds (50 × 50 × 50 mm^3^) when the mixture showed proper viscosity. The specimens were covered with plastic sheets, and those were stored at room temperature for 24 h. Subsequently, the specimens were demolded and cured in a water tank maintained at 23 °C. After 24 h of drying in a laboratory environment, the compressive strength was measured following the ASTM C109 [21] at the age of 28 days. The compressive strengths listed in Table 1 were obtained by averaging the results calculated by measuring three specimens. In addition, in the laser cutting experiment, each specimen was used thrice at different laser cutting speeds. Figure 1 shows the experimental setup and an example of a tested specimen after laser cutting.

The experiment was performed using a 10 kW multimode fiber laser (IPG YLS-10000, IPG Photonics, Oxford, MS, USA) operating at a 1070 nm wavelength. The laser had a spot size of 150 μm, and N_2_ assist gas was maintained at a pressure of 7 bar. The specimen was placed on the testbed and fixed using a vice. The spacing of the testbed was set to 6.5 mm to facilitate the removal of the material discharged during the laser cutting. The focal point was set on the specimen surface, and the laser beam was irradiated vertically onto the specimen during the laser head movement. To collect dust particles during the laser irradiation, a ventilation duct was placed on the left side of laser device. The laser power was set at 9 kW, and the laser cutting speed was designated as the only controllable parameter in laser settings so as to simplify the experimental parameters. The laser cutting speeds were set as 0.25 and 0.5 m/min; in addition, speeds from 1 to 4 m/min in increments of 1 m/min were considered. Table 2 lists the laser cutting speeds and line energies used in the experiment.

**Figure 2:**
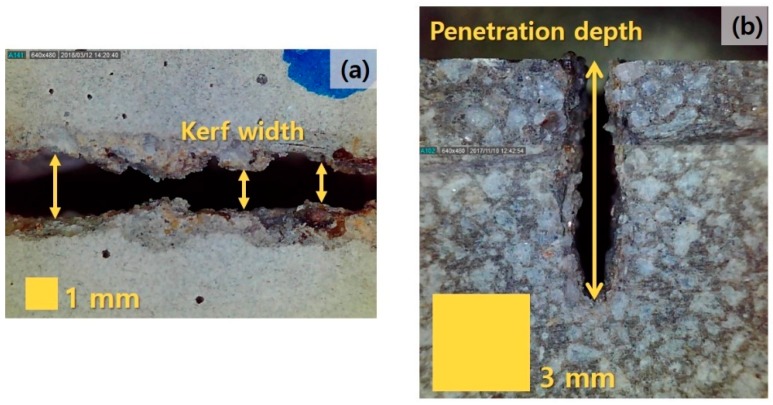
Examples of evaluation of (**a**) kerf width and (**b**) penetration depth.

The line energy, which was obtained by dividing the laser output power by the laser cutting speed and the laser spot size, was used to continuously monitor the irradiated laser energy per unit volume. Furthermore, the line energy was an important parameter used to understand the interaction between the laser and specimens. After the laser cutting (see Figure 2), specimens’ kerf width and penetration depth were observed using a digital microscope (AnMo Electronics Corp., New Taipei, Taiwan).

The kerf width and penetration depth were used to indicate the amount of materials that were removed by the laser processing. That is, the effect of the laser on the materials can be effectively evaluated according to the parameters set in the experiment. The kerf width and penetration depth represented the completely cut width of the specimens at the surface (top surface) and the measured depth of the specimen, respectively, under laser processing. The kerf width was measured at nine points for each case, and the maximum, minimum, and average values of the measured widths were evaluated. In addition, the penetration depth was measured using an additional specimen cut using a mechanical saw cutter with a blade thickness of 3 mm.

## 4. Results and Discussion

### 4.1. Kerf Width

**Figure 3:**
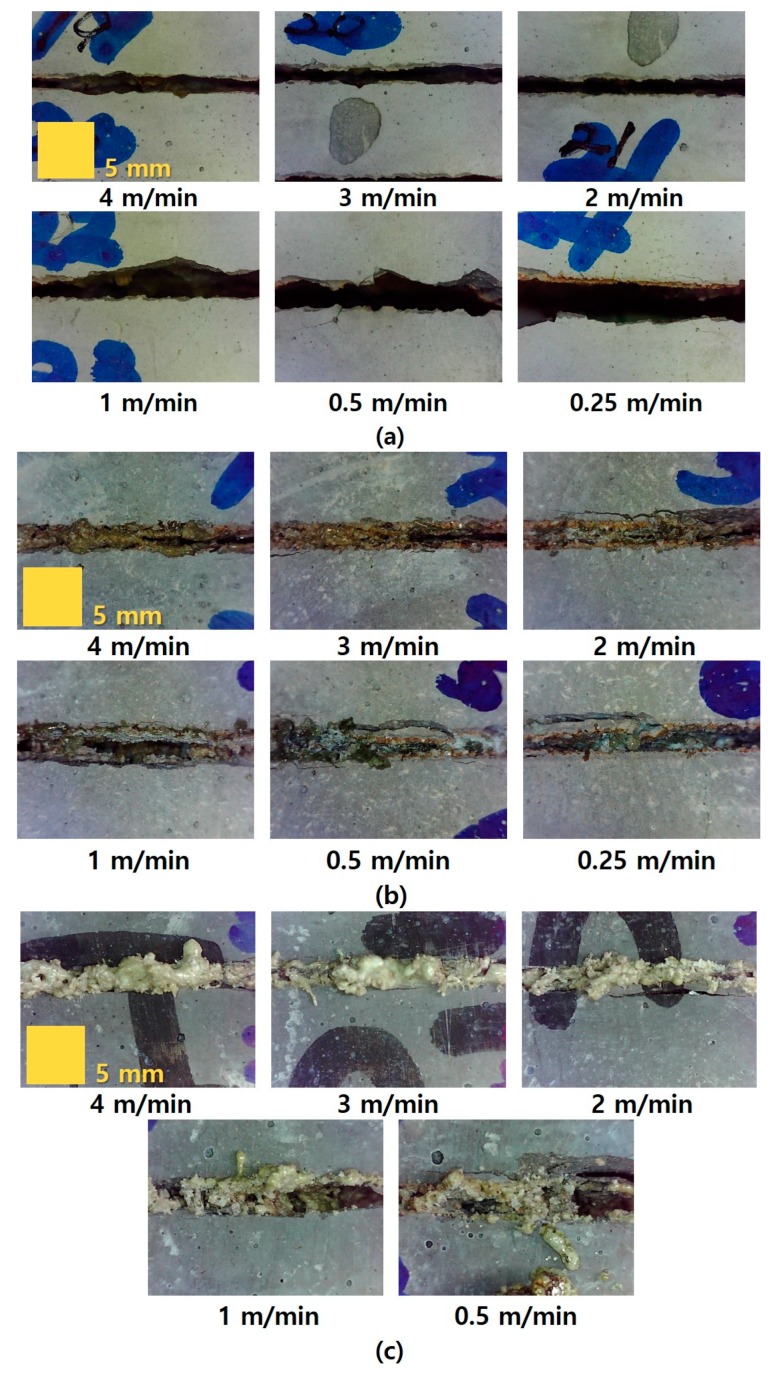
Examples of specimen surfaces after laser interaction: (**a**) LP 0.25, (**b**) LM 0.25, and (**c**) LU-Ⅰ.

Before analyzing the kerf width of each specimen in accordance with the laser cutting speed, the top view of the cement-based materials was observed after the laser irradiation. In this study, the kerf width was set as a characteristic variable. Figure 3 depicts the specimens’ surface of the LP, LM, and LU series within the laser cutting speed range.

**Figure 4:**
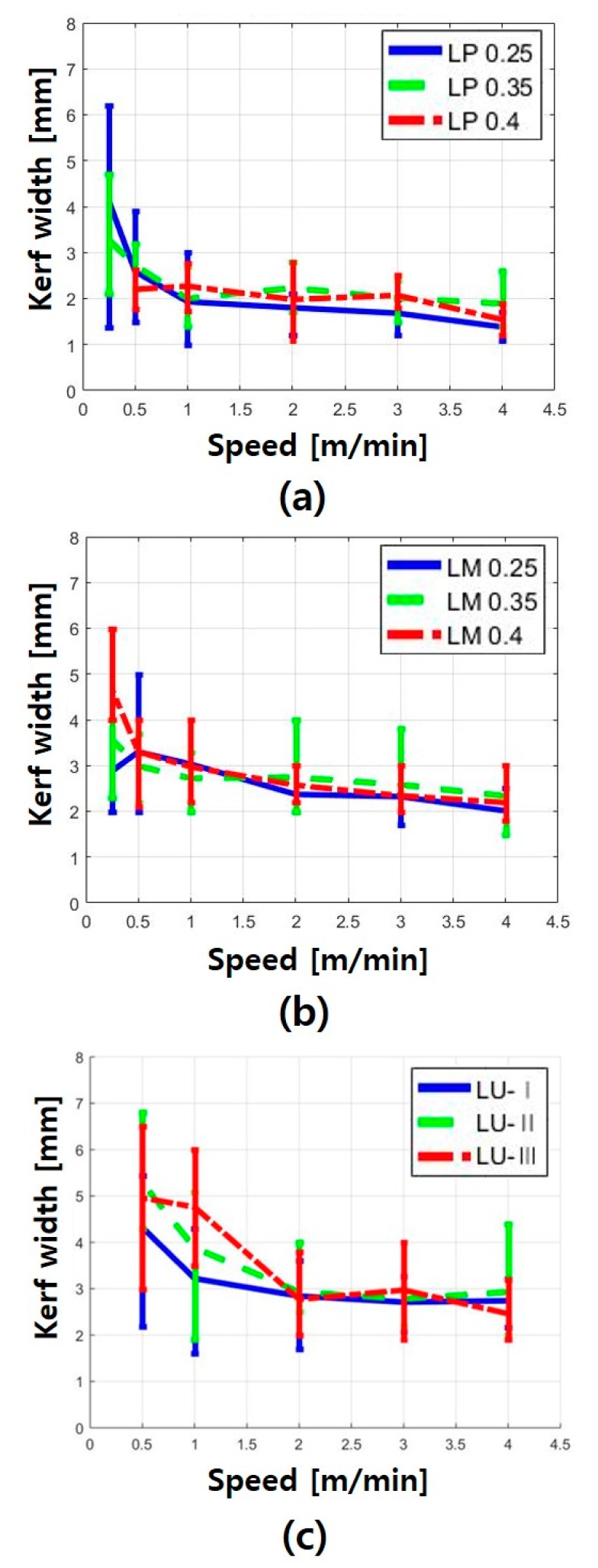
Minimum, maximum, and average values of kerf width on (**a**) LP, (**b**) LM, and (**c**) LU.

It should be noted, that the laser cutting experiment of the LU specimen at the laser cutting speed of 0.25 m/min did not proceed, because the laser nozzle was damaged owing to the generation of inflated consolidated materials during the laser cutting, especially in the case of a very low speed. The kerf width gradually increased with decreasing laser-cutting speed in the entire series. The LP specimen showed good cutting quality, as can be seen in Figure 3a, whereas the LM and LU series showed inflated consolidated materials near the surface. On the surface of the LM specimen, a part of the burnt zone and cracks occurred around the kerf width because of the laser interaction. The impurities expelled from the molten concrete were observed at the surface of the LU specimen. Figure 4 shows the relationship between the kerf width and laser cutting speed for each series in accordance with the cutting speed. The maximum, minimum, and average values of each specimen are also presented. Furthermore, the Y-axis range in all the graphs in Figure 4 is set to be the same, so that the kerf width of each specimen can be easily compared.

**Figure 5:**
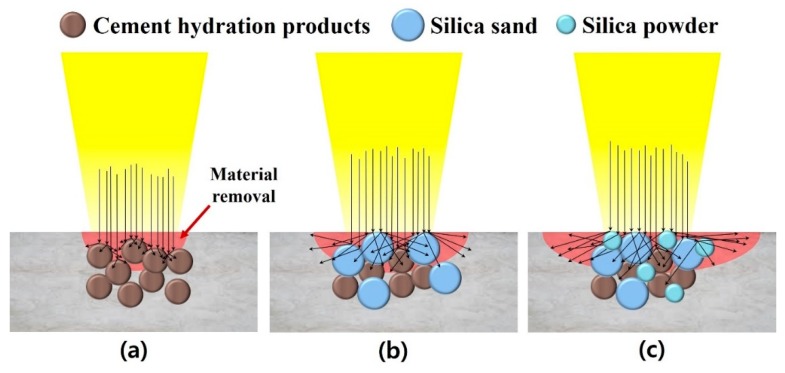
Schematic diagram of material removal mechanisms on (**a**) LP, (**b**) LM, and (**c**) LU.

As the laser cutting speed increased, the kerf width of all the series decreased. LP series showed a lower kerf width than the LM and LU series, as shown in Figure 4. When the cutting speeds were between 0.25 and 1 m/min, the kerf width decreased sharply, but at speeds faster than 1 m/min, the kerf width changed insignificantly. Furthermore, the effect of the water-to-cement ratio on the kerf width in cement paste was not observed clearly. As can be seen in Figure 4b, the LM series had the smallest variation in the kerf width according to the cutting speed. From these results, the effect of silica sand on the cement-based materials was clearly observed. As can be seen in Figure 4c, the variation in the kerf width of the LU series was very irregular. Based on these observations, the material removal mechanism in the cement-based materials was obtained, as shown in the schematic diagram in Figure 5.

In Figure 5, the arrows indicate the reflectance of the laser beam. In addition, the length of the arrows indicates the magnitude of the reflectance. The reflectance of silica sand and silica powder was very high. Thus, a bulk of the laser beam was directed sideways rather than in the downward direction. Consequently, the LM and LU series had a wider kerf width, as shown in Figure 5b,c. Based on this observation, it was assumed that the silicate-based material affected the kerf width of the cement-based materials. Lee and Pyo [22] proposed that the silica sand leads cement-based materials to additional physical changes such as re-solidification, burning, and micro-cracks caused by laser interaction. Since the high pressure decreased during laser irradiation, cracks can generate in small area farther from the re-solidification area.

**Figure 6:**
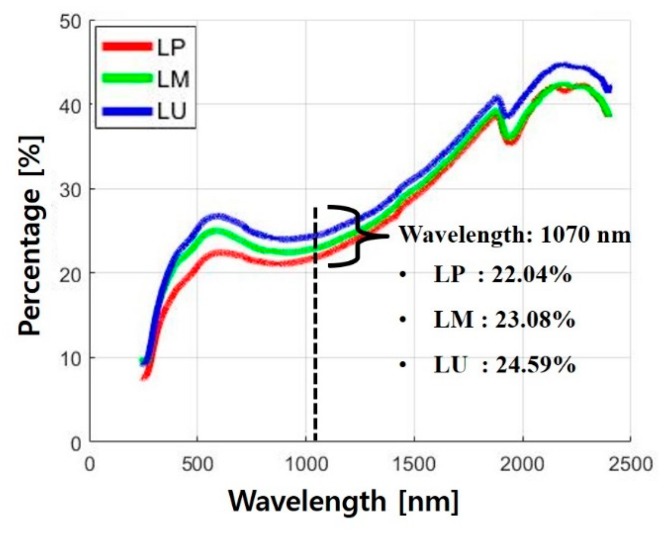
Comparison between diffuse reflectances of cement-based materials.

Figure 6 shows the diffuse reflectances of LP, LM, and LU. The diffuse reflectance was measured by UV–VIS spectrophotometers (SolidSpec-3700, Shimadzu) in the wavelength range from 200 to 2400 nm. The diffuse reflectances of LP, LM, and LU were 22.04%, 23.08%, and 24.59%, respectively, at the wavelength of 1070 nm. As seen in Figure 5, the kerf width increased as the amount of the silicate-based materials increased. This might be explained by the fact that the difference in the diffuse reflectance can be ascribed to the difference in the material removal mechanisms.

### 4.2. Penetration Depth

**Figure 7:**
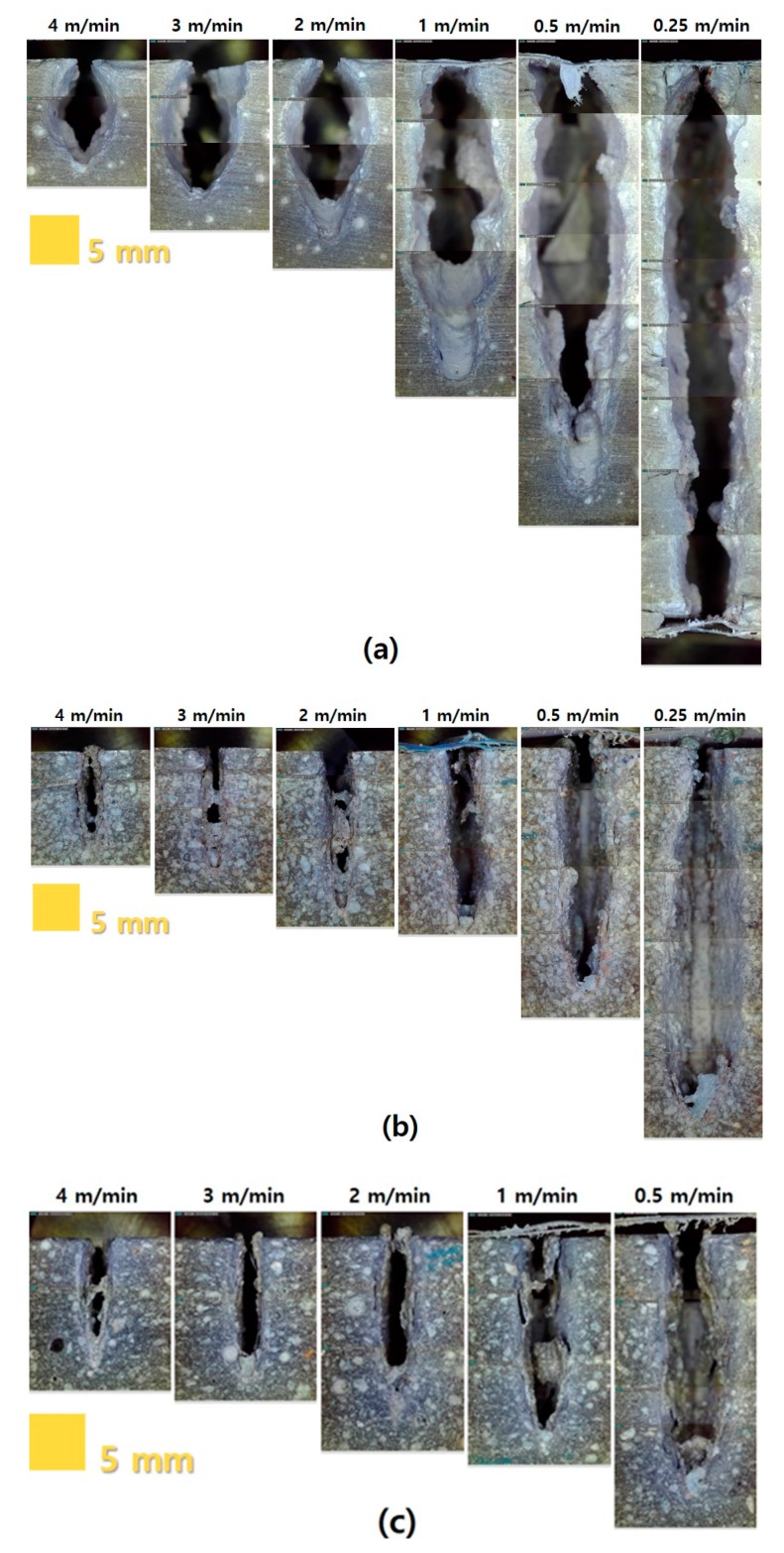
Penetration depth of (**a**) LP 0.25, (**b**) LM 0.25, and (**c**) LU-Ⅰ.

To analyze the penetration depth according to the laser cutting speed, the penetration depth was measured from the concrete surface to the deepest point penetrated by laser processing. In addition, the variation in the depth according to the material composition was mainly observed. Figure 7 shows the cross-sectional views of each series according to the laser cutting speed.

**Figure 8:**
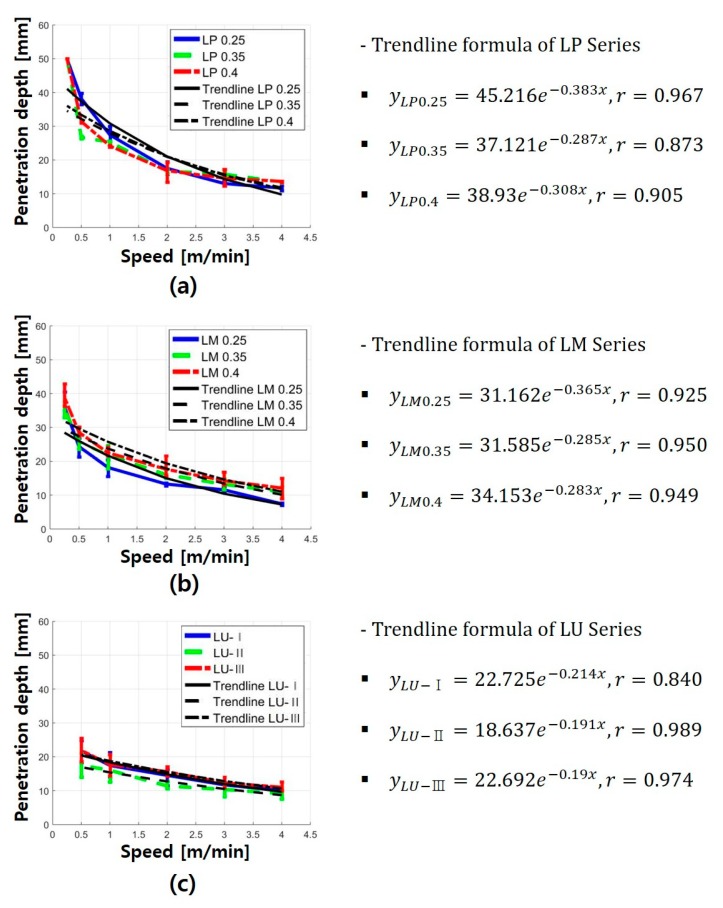
Average penetration depth graph and regression formula of (**a**) LP series, (**b**) LM series, and (**c**) LU series.

The penetration depth of each series increased as the cutting speed decreased. Furthermore, microcracks and macrocracks were observed around the penetration depth. Under the given laser parameters, the penetration depth of the LP series showed a clean material removal zone. In addition, the material removal zone of the LP series was larger than those of the other series. In the LM and LU series, the cement-based materials melted by the laser were not completely removed in the penetrated zone, and impurities were observed on the surface of the cement-based materials. Based on a series of such evaluation processes, relationships between the penetration depth and laser speed were obtained, as summarized in Figure 8. The key variables were the water-to-cement ratio and material composition.

At the laser cutting speed of 4 m/min, the penetration depth was 10–13 mm regardless of the material composition and water-to-cement ratio. However, in the case of the LP series at the speed of 0.25 m/min, full penetration occurred. The line energy required to completely penetrate the LP series with a thickness of 50 mm was observed to be above 1.22 × 10^14^ J/m^3^. In addition, the penetration depth drastically decreased at the cutting speed of 0.25–1 m/min. The LM0.4 specimen showed the maximum penetration depth of 38.85 mm at 0.25 m/min. Furthermore, the penetration depth of the LP and LM series exponentially decreased as the cutting speed increased. However, in the LP and LM series, the effect of the water-to-cement ratio on the penetration depth was not clearly observed. Unlike the LP and LM series, the LU series showed an almost linear relationship with the penetration depth according to the cutting speed. In particular, the overall penetration depth of the LU series was less than 30 mm. This might be attributed to the fact that the compressive strength of the LU series was the highest. Based on this observation, it was assumed that the compressive strength affected the penetration depth of the cement-based materials. Thus, by comparing the LP and LM series, it was concluded that the presence of silica sand led to a decrease in the penetration depth. This was because the strength of the silica sand was considerably higher than that of the cement paste. As shown in Table 1, in addition, the comparison of the LM and LU series indicated that their material strengths were different.

**Figure 9:**
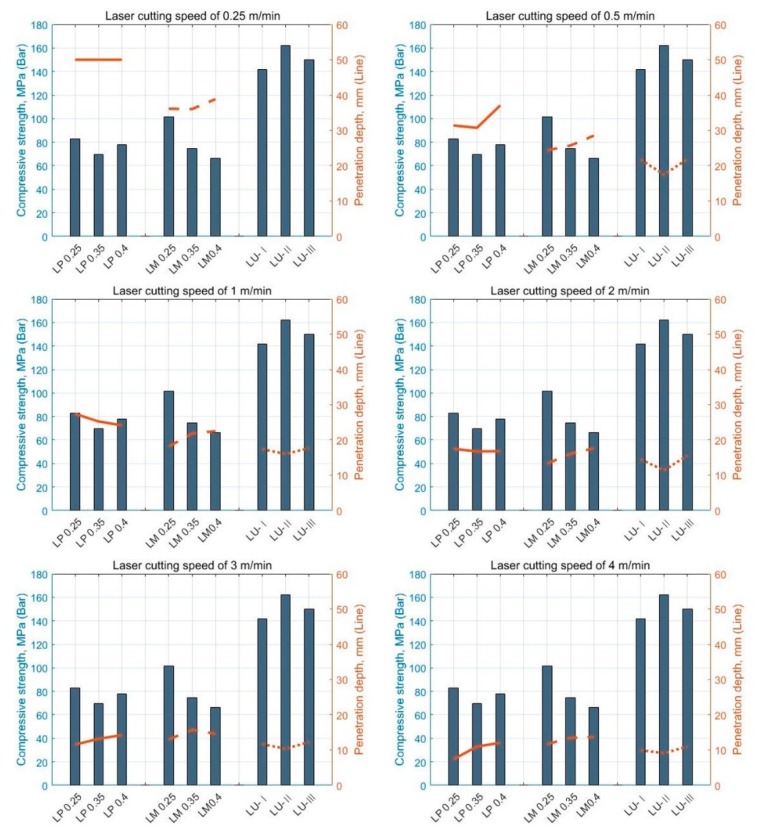
Relationship between compressive strength and penetration depth according to laser cutting speed.

Figure 9 shows the relationship between the compressive strength and penetration depth in accordance with the laser cutting speed. When the laser cutting speed was 0.5 and 1 m/min, as the compressive strength of all the series increased, the overall penetration depth decreased. In addition, differences were clearly observed in the penetration depth for each series. On the other hand, as the laser cutting speed increased beyond 1 m/min, the difference between the penetration depths of LP and LM gradually decreased. However, the LU series showed relatively low variation in the penetration depth with the cutting speed compared to the LP and LM series; this could be the likely cause of the higher compressive strength of the LU series. The compressive strength of the LU series was greater than 140 MPa, which was substantially higher than those of the LP and LM series. This was because the mix proportion of the LU series, i.e., UHPC, was carefully designed based on the particle packing theory to achieve superior mechanical properties unlike conventional cement-based materials. For example, Pyo et al. [23,24] successfully developed new types of UHPC mix designs based on particle packing theory using available solid constituents to achieve outstanding material properties. Therefore, it was concluded that as the compressive strength of cement-based materials increased, the variation in the penetration depth with the laser cutting speed decreased.

### 4.3. Characteristics of Removal Area of LP Series

**Figure 10:**
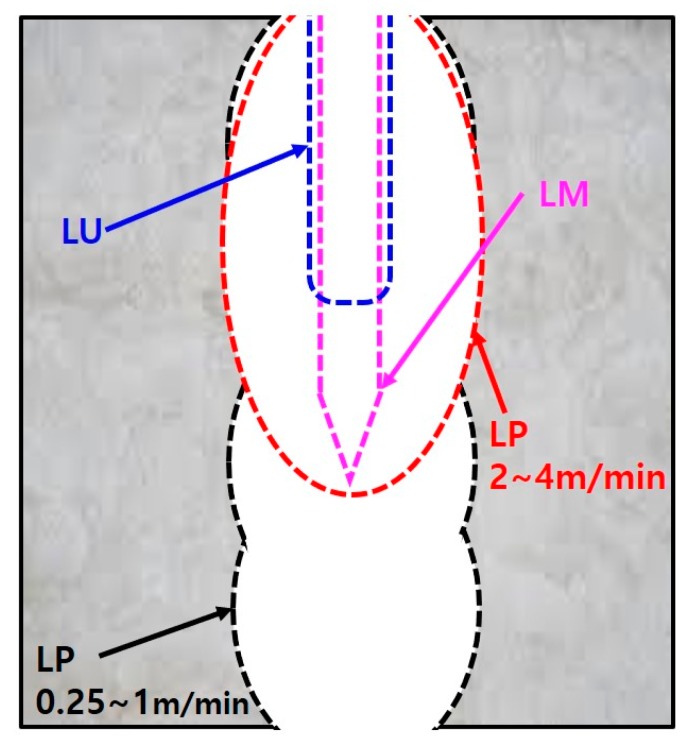
Schematic of penetration shapes of LP, LM, and LU series.

**Figure 11:**
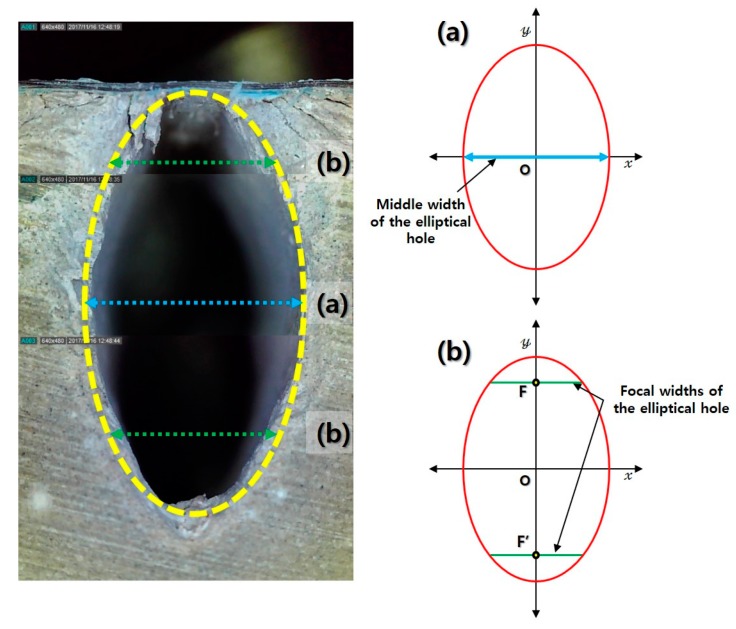
Measurement positions on (**a**) the middle width and (**b**) the focal widths of the elliptical hole.

In the cross-sectional view of each series, the removal characteristics of the material by laser cutting were observed based on the geometry of the penetrated area. Figure 10 shows a schematic of the penetration depth for each series. The general penetration shape of the LM series was nearly rectangular with a pointed end on the bottom side, whereas that of the LU series was rectangular with rounded corners. On the other hand, the penetration shape of the LP series was divided into two parts (single and multiple elliptical shapes) according to the cutting speed. Multiple elliptical shapes described the geometry in which two or more ellipses overlapped in the downward direction. When the cutting speed was 2~4 and 0.25~1 m/min, the penetration shape of the LP series was described by a single elliptical shape and multiple elliptical shapes, respectively. The penetration shape observed for the LP series could be designated as an elliptical hole. Furthermore, the focal widths were calculated using the elliptic equation and compared with the measured widths. Figure 11 shows the calculation of the focal widths in the elliptical hole.

**Figure 12:**
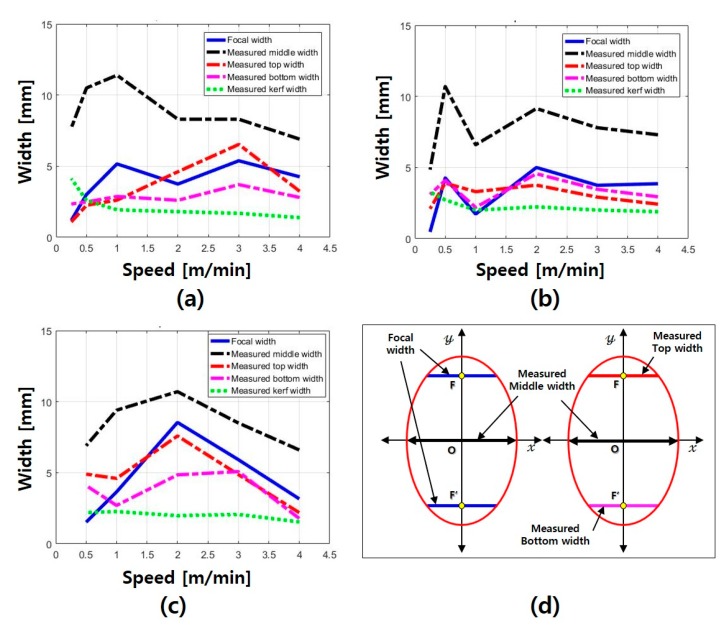
Width comparison for (**a**) LP 0.25, (**b**) LP 0.35, and (**c**) LP 0.4, and (**d**) position of measured and focal widths of elliptical hole.

The focal widths of the elliptical hole (Figure 11b) were calculated using the elliptic equations after designating the measured middle width of the elliptical hole (Figure 11a). The top and bottom widths of the elliptical hole were measured to compare the focal and measured widths. Figure 12 shows the focal and measured widths (kerf, middle, top, and bottom widths) of the LP series according with the cutting speed.

When the LP 0.4 specimen was processed at the speed of 0.25 m/min, the specimen was damaged by heat and could not be measured. In the LP 0.25, LP 0.35, and LP 0.4 specimens, the focal width and measured middle width showed similar variations according to the cutting speed. The focal width and measured middle width were larger than the kerf width. Moreover, most widths of LP 0.25 and LP 0.35 decreased beyond the cutting speed of 3 m/min. In particular, the intervals between the focal and measured widths (top, middle, and bottom widths) gradually decreased. Because of this, it was estimated that the measured and focal widths had the same trend as the cutting speed increased. The maximum measured middle width of the LP series was 11.4 mm, and most widths varied between 6 and 8 mm. Furthermore, when the cutting speed was 0.5 and 4 m/min, the measured middle widths of LP 0.4 were 6.9 and 6.6 mm, respectively. According to these observations, it was estimated that the single elliptical hole was related to the heat transfer of the sidewalls in proportion to the cutting speed. Furthermore, the multiple elliptical holes were estimated to be generated as the absorption rate of the laser was changed by the melted cement paste.

### 4.4. Interaction Mechanism between Laser and Cement-Based Materials

**Figure 13:**
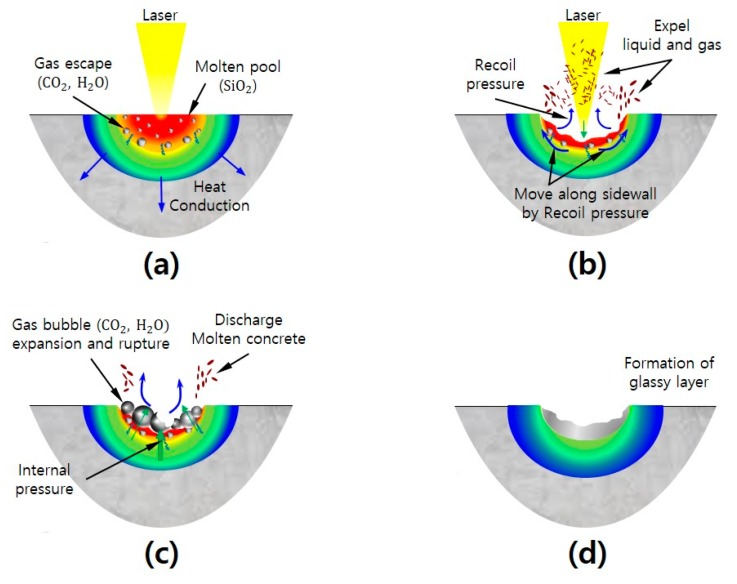
Schematic diagram of interaction mechanism between laser and cement-based materials. (**a**) generation of molten pool by laser; (**b**) discharging the vapor mixed with the liquid; (**c**) creation of gas bubbles from decomposition of cement-based material; (**d**) formation of glass layer from solidification of molten pool.

Based on the experimental results, a schematic diagram of the interaction between the laser and cement-based materials was suggested, as shown in Figure 13. When the laser interacted with the cement-based materials, the surface of the specimen absorbed the laser energy and was heated to a high temperature. Chemical and physical reactions occurred inside the cement-based materials because of the dehydration of calcium hydroxide and the decomposition of calcium carbonate. In this process, gas (CO_2_ and H_2_O) was emitted from the laser irradiation region. When the surface temperature reached 1500–1650 °C, the cement paste and silica sand started to melt. This process created a molten pool (SiO_2_) in the material removal zone, as shown in Figure 13a. When the surface temperature reached the vaporization temperature, some of the molten pool content evaporated. Simultaneously, the molten pool content was blown away by the N_2_ assist gas. A recoil pressure was generated in the molten cement-based materials. Because the recoil pressure was higher than the surface tension of the molten specimen surface, the vapor mixed with the liquid was pushed out of the molten pool. In addition, the recoil pressure expelled the molten pool content along the sidewalls of the hole, as shown in Figure 13b. The material removal zone was maintained at a high temperature after laser irradiation. The released gas produced by the decomposition of the cement-based materials escaped from the high-viscosity layer of the molten pool. The gas produced by the decomposition of the cement-based materials considerably increased the pressure under the molten pool and created a gas bubble on the surface of the molten pool. This process continued until the molten pool was solidified completely. The solidified molten pool formed a glassy layer in the material removal zone. In addition, a glassy layer was formed on the sidewalls and the surface of the material removal zone of the cement-based materials, as shown in Figure 13c,d. It should be noted that the microstructural changes based on the SEM/EDX analysis of the cement-based materials under laser interaction can be found in the authors’ previous research [25].

## 5. Conclusions

This study was conducted to investigate the applicability of laser cutting technology to cement-based materials using multimode fiber lasers. The experimental variation parameters were the material composition and laser cutting speed, which was set to 0.25, 0.5, 1, 2, 3, and 4 m/min. After laser irradiation, the top and cross-sectional views of cement-based materials were observed. Trends were analyzed by measuring the kerf width and penetration depth of the cement-based materials. To elucidate the elliptical hole observed in cross sections of the LP series, the focal and measured widths (top, middle, and bottom widths) were compared through graphs. The major observations and results of this study can be summarized as follows:

1. The kerf width of the LP series showed good cutting quality, but partially cracked and burnt areas were observed in the LM series. In the LU series, not only was the molten pool not completely evaporated in the material removal zone, but impurities were also observed on the surface;
2. Under the considered parameters, the 50-mm-thick cement-based material was completely cut only for the LP series when the cutting speed was 0.25 m/min at 9 kW. To completely cut the 50-mm-thick cement paste (LP series) material, a line energy of 1.22 × 10^14^ J/m^3^ or more was required;
3. Single and multiple elliptical holes were generated by the heat transfer at the sidewalls of the material removal zone and the effect of the molten concrete on the laser absorption rate, respectively.

In the future, as an extension of the present study, the surface temperature should be measured during fast laser cutting for an accurate analysis of the interaction mechanisms between the laser and cement-based materials.

## Figures and Tables

**Table 1:** Cement-based materials mixing design (proportion by weight).

Series	Cement	Water	Silica Fume	Silica Powder	Silica Sand I ^†^	Silica Sand II ^‡^	Superplasticizer ^§^	Compressive Strength (MPa)
LP 0.4	1	0.40	-	-	-	-	-	77.7
LP 0.35	1	0.35	-	-	-	-	-	69.6
LP 0.25	1	0.25	-	-	-	-	-	83.0
LM 0.4	1	0.40	-	-	-	1.0	-	84.7
LM 0.35	1	0.35	-	-	-	1.0	-	87.5
LM 0.25	1	0.25	-	-	-	1.0	0.009	116.3
LU-III	1	0.25	0.15	0.25	0.30	0.70	0.009	150.1
LU-II	1	0.25	0.25	0.15	0.30	0.70	0.009	162.1
LU-I	1	0.25	0.25	0.25	0.30	0.70	0.009	141.6

^†^ Median size = 0.15 mm; ^‡^ Median size = 0.53 mm; ^§^ Solid content.

**Table 2:** Laser speed and line energy used in the experiment.

Index	Speed [m/min]	Line Energy [J/m^3^]
1	4	7.64 × 10^12^
2	3	1.02 × 10^13^
3	2	1.53 × 10^13^
4	1	3.06 × 10^13^
5	0.5	6.11 × 10^13^
6	0.25	1.22 × 10^14^

## References

[1] Moon S., Yang B., Kim J., Seo J. (2010). Effectiveness of Remote Control for a Concrete Surface Grinding Machine. Autom. Constr..

[2] Skarabis J., Stöckert U. (2015). Noise Emission of Concrete Pavement Surfaces Produced by Diamond Grinding. J. Traffic Transp. Eng..

[3] Mostavi E., Asadi S., Ugochukwu E. (2015). Feasibility Study of the Potential Use of Drill Cuttings in Concrete. Procedia Eng..

[4] Foroutan M., Hassan M.M., Desrosiers N., Rupnow T. (2018). Evaluation of the Reuse and Recycling of Drill Cuttings in Concrete Applications. Constr. Build. Mater..

[5] Sitek L., Bodnárová L., Válek J., Zeleňák M., Klich J., Foldyna J., Novotný M. (2013). Effects of Water Jet on Heat-Affected Concretes. Procedia Eng..

[6] Lee D., Oh B., Suk J. (2019). The Effect of Compactness on Laser Cutting of Cathode for Lithium-Ion Batteries Using Continuous Fiber Laser. Appl. Sci..

[7] Wetzig A., Herwig P., Hauptmann J., Baumann R., Rauscher P., Schlosser M., Pinder T., Leyens C. (2019). Fast Laser Cutting of Thin Metal. Procedia Manuf..

[8] Pocorni J., Powell J., Deichsel E., Frostevarg J., Kaplan A.F.H. (2017). Fibre Laser Cutting Stainless Steel: Fluid Dynamics and Cut Front Morphology. Opt. Laser Technol..

[9] Sun M., Eppelt U., Hartmann C., Schulz W., Zhu J., Lin Z. (2016). Damage Morphology and Mechanism in Ablation Cutting of Thin Glass Sheets with Picosecond Pulsed Lasers. Opt. Laser Technol..

[10] Lee D., Cho J., Kim C.H., Lee S.H. (2017). Application of Laser Spot Cutting on Spring Contact Probe for Semiconductor Package Inspection. Opt. Laser Technol..

[11] Haddadi E., Moradi M., Ghavidel A.K., Ghavidel A.K., Meiabadi S. (2019). Experimental and Parametric Evaluation of Cut Quality Characteristics in CO_2_ Laser Cutting of Polystyrene. Optik.

[12] Muto S., Tei K., Fujioka T. (2007). Laser Cutting for Thick Concrete by Multi-Pass Technique. Chin. Opt. Lett..

[13] Lee D., Seo Y., Pyo S. (2018). Effect of Laser Speed on Cutting Characteristics of Cement-Based Materials. Materials.

[14] Crouse P.L., Li L., Spencer J.T. (2004). Performance Comparison of CO_2_ and Diode Lasers for Deep-Section Concrete Cutting. Thin Solid Films.

[15] Pyo S., El-Tawil S. (2015). Capturing the strain hardening and softening responses of cementitious composites subjected to impact loading. Constr. Build. Mater..

[16] Pyo S., Alkaysi M., El-Tawil S. (2016). Crack propagation speed in ultra high performance concrete (UHPC). Constr. Build. Mater..

[17] Kim H., Koh T., Pyo S. (2016). Enhancing flowability and sustainability of ultra high performance concrete incorporating high replacement levels of industrial slags. Constr. Build. Mater..

[18] Pyo S., Abate S.Y., Kim H.K. (2018). Abrasion resistance of ultra high performance concrete incorporating coarser aggregate. Constr. Build. Mater..

[19] Pyo S., Tafesse M., Kim H., Kim H.K. (2017). Effect of chloride content on mechanical properties of ultra high performance concrete. Cem. Concr. Compos..

[20] Pyo S., Tafesse M., Kim B.J., Kim H.K. (2018). Effects of quartz-based mine tailings on characteristics and leaching behavior of ultra-high performance concrete. Constr. Build. Mater..

[21] (2016). ASTM C109/C109M-16a, Standard Test Method for Compressive Strength of Hydraulic Cement Mortars (Using 2-in. or [50-mm] Cube Specimens).

[22] Lee D., Pyo S. (2018). Experimental Investigation of Multi-Mode Fiber Laser Cutting of Cement Mortar. Materials.

[23] Pyo S., Kim H.K., Lee B.Y. (2017). Effects of Coarser Fine Aggregate on Tensile Properties of Ultra High Performance Concrete. Cem. Concr. Compos..

[24] Pyo S., Kim H.K. (2017). Fresh and hardened properties of ultra-high performance concrete incorporating coal bottom ash and slag powder. Constr. Build. Mater..

[25] Seo Y., Lee D., Pyo S. (2020). Microstructural characteristics of cement-based materials fabricated using multi-mode fiber laser. Materials.

